# Management of stage one and two-E gastric large B-cell lymphoma: chemotherapy alone or surgery followed by chemotherapy?

**DOI:** 10.1186/1756-8722-3-23

**Published:** 2010-06-22

**Authors:** Yassir Sbitti, Nabil Ismaili, Youssef Bensouda, Habiba Kadiri, Mohammed Ichou, Hassan Errihani

**Affiliations:** 1 Department of Medical Oncology, Mohammed V Military Hospital, Rabat, Morocco; 2 Department of Medical Oncology, National Institute Hospital of Oncology, Rabat Morocco; 3 Department of Pathology, National Institute Hospital of Oncology, Rabat Morocco

## Abstract

Management of localized primary gastric B lymphoma (PGL) remains controversial. The aim of this study is to compare two treatments: chemotherapy alone and surgery plus chemotherapy.

**Materials:**

Records of all patients with a diagnosis of gastric lymphoma and which were treated in the National Institute of Oncology, between 1999 and 2006, were reviewed and patients fulfilling the following criteria were included in this study: histologically proven large-cell B lymphoma of the stomach; complete clinical information stage I/II disease according to the Musshoff staging; patients who received surgery followed by chemotherapy (group I) or chemotherapy alone (group II).

**Results:**

This study included 82 patients who were treated for cancer in our Institute. All clinical and pathological features were similar between the two groups, except that patients of group-I had significantly more stage II disease (P = 0.023) than that of group II. Among the 52 patients who could be evaluated for response to chemotherapy, there were 45 who had complete response to treatment, 3 had partial response to the treatment and 4 had progressive disease. The projected 5-year relapse-free survival (RFS) and overall survival (OS) of group I were 86.69% (95% CI, 57.9 - 97.7%) and 90.0% (95% CI, 58.0 - 97.8%), respectively. And the projected 5-year relapse-free survival RFS and OS of group II were 86.67% (95% CI, 57.0 - 88.2%) and 93.33% (95% CI, 73.3 - 98.7%) respectively. There were no statistically significant differences in RFS (P = 0.485) and OS (P = 0.551) between the two groups.

**Conclusion:**

Our data suggest that chemotherapy alone may be a reasonable alternative treatment for stage I/II gastric large-cell lymphoma but this result must be confirmed by prospective randomized clinical trials.

## Introduction

Surgery has been the conventional treatment for patients with localized gastric lymphoma [1,2]. Adjuvant chemotherapy or radiotherapy was often used for patients with regional lymph node involvement. Systemic chemotherapy has been the treatment of choice for most nodal and extra nodal lymphomas as reported in published data which support the safety and efficacy of conservative treatments in the case of stage I/II primary gastric large-cell B lymphoma (PGDLCL). As the primary chemotherapy treatment was given either alone or followed by radiation therapy, the role of surgical resection of the primary tumor needs to be clearly defined and justified [3-5]. This retrospective study investigated the clinical outcome of localized gastric lymphoma treated by chemotherapy alone or surgery followed by chemotherapy.

## Methods

### Patients

All records of patients which were diagnosed as having gastric lymphoma during the period 1999 and 2006, were reviewed and patients fulfilling the following criteria were included in this study: histological proven large-cell B lymphoma of the stomach; complete clinical information for stage I/II disease (Musshoff modification of Ann Arbor system); patients who received curative surgery followed by adjuvant anthracycline based chemotherapy (group I) or chemotherapy alone with anthracycline-containing regimens (group II), primary management and follow up in our institution. Patients with mucosa associated lymphoid tissue (MALT) lymphoma were excluded.

### Clinical evaluation

Staging procedures included complete physical examinations, inspection for waldeyer's ring, complete blood cell count and differential count, blood chemistry, upper gastrointestinal endoscopy, chest and abdomen CT scan, bone marrow aspiration and biopsy. The staging was determined according to the Musshoff modification of Ann Arbor system [6] which divided stage II disease into stage IIE1 and stage IIE2. In stage IE the tumor remains confined within the stomach; in stage IIE1 the perigastric nodal involvement was positif; in stage IIE2 more distant nodal involvement was found up to the region below the diaphragm. Grading of treatment toxicity as well as tumor response was evaluated according to the criteria defined by the World Health Organization [7]. Response to chemotherapy was evaluated by physical examination, endoscopy, and image studies every 3 cycle of chemotherapy. Complete response (CR) was defined as the disappearance of all evidence of tumor(s) for a duration of at least 4 weeks. Partial response (PR) was defined as > 50% reduction in the sum of the products of the longest perpendicular diameters of all measurable lesions in radiographic images, with the reduction lasting at least 4 weeks. Stable disease (SD) was defined as < 50% reduction or < 25% increase in the sum of the products of the longest perpendicular diameters of all measurable lesions, lasting > 4 weeks. Patients with progressive lesions were not classified as having PR or SD. Progressive disease (PD) was defined as the appearance of new lesions or > 25% increase in the area(s) of original measurable disease.

### Statistical analysis (SPSS16.0)

Comparisons between clinical and pathological features were done by Pearson chi-square test. Overall survival was calculated from the date of diagnosis to the date of last follow-up or death from any cause. Relapse-free survival was calculated from the date of surgery for group I or complete remission for group II to the date of tumor relapse defined by the results of imaging studies or endoscopic biopsy. Survival distribution of relapse free survival and overall survival were plotted by the estimating method of Kaplan and Meier [8]. Different survival curves were compared with the log-rank test. All *p *values were two-tailed and a *p *value < 0.05 was considered to be statistically significant. SPSS version 16.0 was used for all statistical analyses.

### Consent and statement of ethical approval

As the treatment of each patient was decided by the medical staff of the centre, oral consent was obtained from the subjects and was approved by the institutional review boards of the National Institute of Oncology, Cancer Centre in Rabat

This study was approved by the institutional review boards of National Institute of Oncology, in Rabat.

## Results

### Patients Characteristics

Patients' characteristics are summarized in table 1. Eighty-two patients who fulfilled the broad-spectrum diagnostic criteria for PGL, excluding those with MALT lymphoma, were identified. Among 82 patients, 52 who received chemotherapy alone were categorized into the group II and the other 30 who received total gastrectomy followed by chemotherapy were categorized into group I. Clinico-pathological features of the patients are listed in table 1. No significant difference was noted for all other major characteristics between these two groups. Group II had significantly more localized disease with fewer patients in stage II-2 (p = 0.023). All patients received Anti-ulcer therapy during chemotherapy.

**Table 1:** Characteristics of patients with localized and advanced primary gastric lymphoma treated with surgery followed by chemotherapy or chemotherapy alone

Parameters	Surgery plus chemotherapy Group I (n = 30) (%)	Chemotherapy Group II (n = 52) (%)	*P *value
Age years	52	53	
Range	19-79	19-81	

Sexe			
Male	25 (83%)	33 (64%)	*P *= 0.057
Female	5 (17%)	19 (36%)	

Musshoff			
Staging			
I	26 (86%)	32 (61%)	*P *= 0.023
II-1	3 (10%)	6 (12%)	
II-2	1 (4%)	14 (27%)	

### Response to Treatment

All patients received CHOP (cyclophosphamide, doxorubicin, vincristine, prednisone) chemotherapy regimen which consisted of intravenous injection of cyclophosphamide 750 mg/m², doxorubicin 50 mg/m², and vincristine 1.4 mg/m² (maximum 2 mg) on day 1, and prednisone 60 mg/m2 orally on days 1-5. The median number of cycles of chemotherapy was 4 (range: from 1 to 6) for group I, and 5 (range: from 3 to 8) for group II. For group I, thirteen patients underwent total gastrectomy with curative intent before chemotherapy. Among those patients we evaluated the response to chemotherapy alone in group II in which complete response was achieved in 87% (45/52), partial responses in 6% and progression disease in 7%. Salvage gastrectomy was undergone for five patients: three had gastric perforation and two had upper gastrointestinal bleeding.

### Outcome of the patients

**Figure 1:**
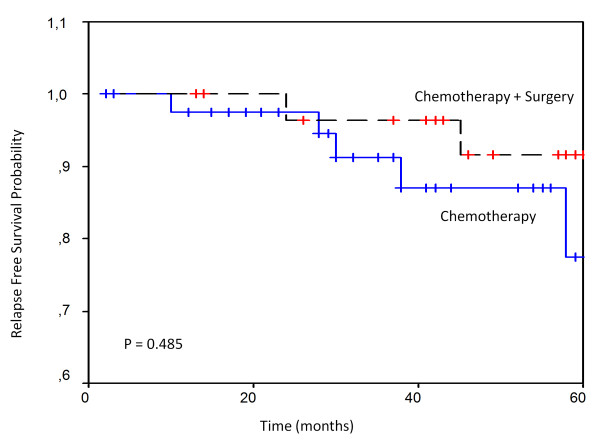
**Relapse-free survival of localized primary gastric lymphoma**.

**Figure 2:**
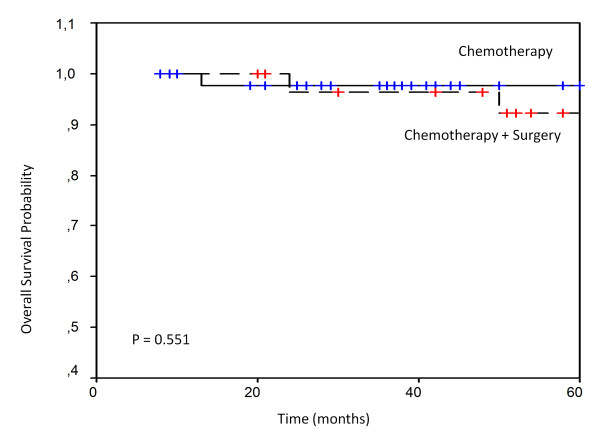
**Overall survival of localized primary gastric lymphoma**.

Only one local relapse occurred in chemotherapy group II and the others relapses in the 2 groups were disseminated. The projected 5-year RFS and OS of group I were 86.69% (95% CI, 57.9 - 97. 7%) and 90.0% (95% CI, 58.0 - 97.8%) respectively. The projected 5-year relapse-free survival (RFS) and overall survival (OS) were 86.67% (95% CI, 57.0 - 88.2%) and 93.33% (95% CI, 73.3-98.7%) respectively in group II. There were no statistically significant differences in RFS (P = 0.485) and OS (P = 0.551) between the two groups (figure 1 and 2).

### Treatment-Related Toxicities

The treatment-related toxicities are summarized in table 2. There were no treatment-related deaths. Grade 3/4 leukopenia was the most side effects for group II. Adjuvant chemotherapy for group I result in similar incidence of haematological toxicity. After administration of chemotherapy in group II, three patients (one patient after first cycle and two after two cycles) developed gastric perforation and two patients (after first cycle) gastrointestinal bleeding. Both of these complications were successfully managed by surgical emergency repair. All these patients presented with stage II-2 disease, with performance status (PS) 2/3, and older age 72 years (range: 67-81 years). They died of distance disease progression after 6, 13 and 18 months after diagnosis respectively. Anastomosis leakage was noted in two patients in group I. They had poor PS (3/4), weight loss (20%) and dismal nutritional status. They died after septic choc.

**Table 2:** Treatment-related complications of primary gastric lymphoma

	Group I		Group II	
	
Toxicity	Nb	%	Nb	%
Leucopoeniae (G3/4)	5	15	10	18

Thrombocytopeniae (G3/4)	1	0.3	0	0

Fever	2	0.6	3	0.5

UGI Bleeding	NA	NA	2	0.3

Anastomosis leakage	2	0.03	NA	NA
Gastric obstruction	NA	NA	1	1.9%
perforation	NA	NA	3	5.7%

Abbreviations. G: grade; UGI: upper gastrointestinal; NA: not applicable

## Discussion

This retrospective study suggests that the clinical outcome of localized PGL treated by chemotherapy alone is comparable to that treated by surgery combined with chemotherapy in terms of disease-free survival and overall survival, so surgery is not required. Review of the literature showed that most of the relevant studies of treatment and outcome of PGL, considered small numbers of patients and were conducted retrospectively [9,10]. The optimal treatment for localized PGL remains to be established. Earlier studies claimed that surgery was the first-line treatment of choice for patients with localized gastric lymphoma [11,12]. Advocates for primary surgery included that patients who underwent surgery had a better survival than those who did not, and surgery might reduce the risk of bleeding or perforation during chemotherapy or radiotherapy. However because the success of surgical management of PGL depends on tumor size, the depth of its penetration into gastric tissue, and the involvement of regional lymph nodes [13-15] some investigators began using chemotherapy, mostly CHOP and its related regimens, to control the tumors and prevent postoperative morbidity gastrectomy [9,16,17]. Recently the roles of stomach-conserving therapies for localized PGL have been emphasized. Relatively little data, however, exist for chemotherapy as sole treatment modality in localised gastric DLBCL, which nevertheless are highly promising and suggest that combination therapy might over treat a substantial proportion of patients [3,5]. Maor and al showed that the 6-year overall survival of patients treated with chemotherapy alone was 76% [17]. However, for bulky tumors, the advantage of chemotherapy is overshadowed by the potential for tumor bleeding and gastric perforation. Most studies have revealed a rather low incidence of severe haemorrhage or perforation, accounting for 2.1% and 1.7%, respectively, of those individuals treated with chemotherapy alone, and 2.2% and 0.9%, respectively, of surgically-treated individuals [17,18]. Such evidence suggests that the role of surgery in the treatment of PGL may be less important than previously considered. In our study, gastric perforation and gastric bleeding developed respectively in 3 patients and 2 patients receiving primary chemotherapy and thus this remains a real and noteworthy complication. To avoid such severe complications, we recommend re-evaluating patients by endoscopy after two cycles of chemotherapy. At the same time, patients should be warned that complications such as gastric perforation and bleeding are possible, and awareness programs involving comprehensive education should be part of the treatment process [19]. Our study has provided good evidence in support of chemotherapy alone. The best management of PGL has yet not been established and the choice of treatment modality is mainly dependent on the expertise of the primary responsible specialists. Oncologists preferred systemic chemotherapy alone and reserved surgery as salvage treatment, while surgeons preferred curative resection followed by adjuvant chemotherapy [20]. Such variation in patient selection has made comparison among different studies difficult. Prospective studies are needed to evaluate each strategy in terms of both survival and treatment-related complications. Our data suggest that systemic chemotherapy alone may be a reasonable alternative treatment for stage I/II large-cell lymphoma of the stomach. We may presume, however, that organ function is better preserved by chemotherapy alone than surgery. Resection of the primary tumor before systemic chemotherapy does not appear to improve the cure rate of this group of patients and could be reserved for those with severe complication (severe bleeding or perforation) after chemotherapy but this result must be confirmed in prospective randomized clinical trial including monoclonal antibody.

## Competing interests

The authors declare that they have no competing interests.

## Authors' contributions

YS: Analysis and collected data, designed study and drafted the manuscript

NI: participated in the design of the study, in the statistical analysis, and helped to draft the manuscript and review of the final manuscript

YB and HK: conceived of the study, and participated in its design and coordination.

HE and MI: review of the final manuscript and revising it critically for important intellectual content.

All authors read and approved the final manuscript.
